# Association of Erie Railway Surgeons

**Published:** 1892-08

**Authors:** 


### ASSOCIATION OF ERIE RAILWAY SURGEONS.

The semi-annual meeting of the Association of the Erie Railway Surgeons was held at the Tifft House, in Buffalo, Tuesday, July 12, 1892. The Association was organized in Elmira last January, and includes all surgeons doing work on the Erie lines between New York and Chicago.

The following-named members were present: President, C. M. Daniels, of Buffalo; Vice-President, R. S. Harnden, Waverly; Drs. W. W. Appley, Cochecton; W. V. R. Blighton, Tonawanda; E. Griswold, Sharon, Pa.; W. B. Hubbs, Addison; John L. Eddy, Olean; M. D. Ellison, C. N. Bradsted, C. S. Parkill, M. J. Baker, B. C. Wakely, Charles R. Phillips, J. G. Kelly, Hornellsville; A. H.

Smith, Avon; William R. Blakeslee, Forest City, Pa.; Henry S. Durand, Rochester; J. E. Janes, Newark, N. J.; D. B. Van Wagenen, Suffern; A. E. Jenner, Dayton, O.; Fred C. Beals, Salamanca; S. L. Sweeney, Newburg, C. B. Kibler, Corry, Pa.; A. G. Ellinwood, Attica; E. M. Dooley, L. J. McAdam, Buffalo; George N. Hall, Binghamton; Charles G. R. Jennings, Henry Flood, Elmira; J. D. Zwetsch, Gowanda; Samuel Birdsall, Susquehanna, Pa.; James B. Stewart, Bradford, Pa.; George D. Crandall, Blossburg, Pa.; Henry A. Argue, Corning; and N. B. Johnson, Shohola.

Papers were read as follows: Traumatic Tetanus, A. G. Ellinwood, M. D., Attica, N. Y.; Surgical Dressings as Applied to Railway Injuries, J. G. Kelley, M. D., Hornellsville, N. Y.; Suggestions upon the Management of Railway Injuries and Modern Antiseptic Treatment, R. S. Harnden, M. D., Waverly, N. Y.; Progressive Surgery, George Chaffee, M. D., Brooklyn, N. Y.; Treatment of Fractures of the Humerus at the Elbow Joint, Henry Flood, M. D., Elmira, N. Y.; Surgery, Now and Then, E. Y. King, Richmond, O.; Restoration of Function in Contused Nerves, John P. Sawyer, M. D., Cleveland, O.; A Case of Fracture of the Skull, Charles G. R. Jennings, M. D., Elmira, N. Y.; Injuries to the Ankle, Webb J. Kelly, M. D., Galion, O.; Three Requisite G's for a Railway Surgeon, J. E. Janes, M. D., Newark, N. J.; Ins and Outs of the Profession, A. E. Jenner, M. D., Dayton, O.; Locomotor Ataxia, J. S. Dolson, M. D., Hornellsville, N. Y.

After the transaction of some routine business, and the adoption of a memorial relating to the death of Dr. W. T. Green, of Hornellsville, the meeting adjourned.

Cleveland was chosen as the next place of meeting; time, January 5, 1893.

---

Resorcin, one of the phenol derivatives, has stood the test of time remarkably well. Its most general use has been in skin affections, but good results have been obtained in whooping-cough and diphtheria, and in checking vomiting. Its anodyne effects may be increased until it acts, in large doses, as a hypnotic. Its antizymotic effects have proved very marked. There has been, recently, an increasing use in the treatment of the gastroenteritis of children. It apparently arrests fermentation in the alimentary canal. It has an advantage over calomel, when used in this affection, by not increasing cardiac weakness. It has repeatedly given good results in cases of septic poisoning from post-mortem examinations. It has now been long enough on trial to become officinal.—*Ephemeris.*

